# Model-based approach for predicting the impact of genetic modifications on product yield in biopharmaceutical manufacturing—Application to influenza vaccine production

**DOI:** 10.1371/journal.pcbi.1007810

**Published:** 2020-06-29

**Authors:** Stefanie Duvigneau, Robert Dürr, Tanja Laske, Mandy Bachmann, Melanie Dostert, Achim Kienle

**Affiliations:** 1 Institute for Automation Engineering, Otto von Guericke University, Magdeburg, Saxony-Anhalt, Germany; 2 Process Synthesis and Process Dynamics, Max Planck Institute for Dynamics of Complex Technical Systems, Magdeburg, Saxony-Anhalt, Germany; 3 Bioprocess Engineering, Max Planck Institute for Dynamics of Complex Technical Systems, Magdeburg, Saxony-Anhalt, Germany; University of California Riverside, UNITED STATES

## Abstract

A large group of biopharmaceuticals is produced in cell lines. The yield of such products can be increased by genetic engineering of the corresponding cell lines. The prediction of promising genetic modifications by mathematical modeling is a valuable tool to facilitate experimental screening. Besides information on the intracellular kinetics and genetic modifications the mathematical model has to account for ubiquitous cell-to-cell variability. In this contribution, we establish a novel model-based methodology for influenza vaccine production in cell lines with overexpressed genes. The manipulation of the expression level of genes coding for host cell factors relevant for virus replication is achieved by lentiviral transduction. Since lentiviral transduction causes increased cell-to-cell variability due to different copy numbers and integration sites of the gene constructs we use a population balance modeling approach to account for this heterogeneity in terms of intracellular viral components and distributed kinetic parameters. The latter are estimated from experimental data of intracellular viral RNA levels and virus titers of infection experiments using cells overexpressing a single host cell gene. For experiments with cells overexpressing multiple host cell genes, only final virus titers were measured and thus, no direct estimation of the parameter distributions was possible. Instead, we evaluate four different computational strategies to infer these from single gene parameter sets. Finally, the best computational strategy is used to predict the most promising candidates for future modifications that show the highest potential for an increased virus yield in a combinatorial study. As expected, there is a trend to higher yields the more modifications are included.

## Author summary

In the present work, we use a sophisticated simulation-based methodology to account for the impact of genetic modifications in producer cell lines on the yield of biomanufacturing processes. Furthermore, our approach opens the possibility to predict the most promising genetic modifications instead of identifying them in costly and time-consuming screening experiments. As an example, we apply our methodology to cell culture-based influenza vaccine production, a process that is of tremendous importance for the maintenance of public health. Here, we consider cell lines in which genes coding for one or more cellular factors are up-regulated by genetic engineering to increase the virus yield. However, the gene editing procedure increases the heterogeneity in the producer cell population because genetic modifications do not occur equally in each cell. This cell-to-cell variability is taken into account in a population balance modeling framework, thus providing a more accurate prediction of the virus yield in the heterogeneous population. Finally, we use our approach and a concise experimental data set from cell lines with one gene modification to predict the virus yield of cell lines with multiple genetic modifications. Thereby, we facilitate the experimental screening of potential candidates. We suggest that this methodology is transferable to a wide range of biomanufacturing processes and constitutes a valuable contribution to experimental design.

## Introduction

Today, a wide range of biopharmaceutical products, e.g. recombinant proteins and viral biopharmaceuticals are produced in cell lines [1]. While for recombinant protein production, process yield is mainly limited by the transcriptional and translational capacity of the cell, the manufacturing of viruses can provide additional burden to the producer cell. In particular, virus-induced changes, related to cell death or to anti-viral signalling, further hamper the process yield. To overcome this limitation, one option is to manipulate the expression level of host cell factors (HCFs) relevant for virus replication in order to enhance virus yield. However, to identify promising HCF candidates costly and time-consuming experimental screening and validation studies are required, as shown recently for poliovirus [2, 3]. This motivates the development of suitable computational tools to predict the impact of genetic modifications in face of the inevitable cellular heterogeneity on the overall product yield. Thereby, promising candidates can be chosen from computational studies decreasing the number of screening experiments which results in a faster and less costly process development. In this study we apply such a methodology to overcome bottlenecks in cell culture-based influenza vaccine production by using genetically modified cell lines.

The influenza virus is responsible for triggering severe pandemics as the Spanish Flu in 1918 which caused a large number of deaths worldwide [4]. Today, the best measure to limit the spread of the virus and thus, avoid epidemic and pandemic outbreaks is vaccination against seasonal strains. These strains are predicted by the World Health Organization every year. In particular, in case of a pandemic outbreak a fast adaption of the vaccine production process becomes necessary to guarantee full protection against influenza. Besides the conventional egg-based production processes, cell culture-based processes are a promising option to produce virus particles in a flexible and reliable manner. Therein, the virus utilizes the transcription and translation machinery of the host cells to replicate itself.

So far, in the literature a series of experimental studies can be found, which focus on HCFs affecting the viral replication cycle in a positive or negative way resulting in an increased or decreased virus yield [5–9]. Based on this knowledge, engineering of novel producer cell lines by overexpressing the genes of selected HCFs is a promising option to improve the production process. One method to overexpress specific genes is lentiviral transduction [11]. In addition to cell lines with single gene overexpression (SGOs), cell lines with multiple gene overexpressions (MGOs) can be generated with this technique, which hold the promise of achieving even higher virus yields. However, in advance it is not immediately evident which combination will provide the desired output and a large number of MGO candidates would have to be screened in time-consuming and expensive experiments. Hence, computational tools are required to help predicting the most promising MGO candidates and thereby support future experimental design and guide MGO generation by lentiviral transduction.

Since lentiviral transduction is a non-targeted technique, copy numbers and integration sites of gene constructs vary between individual cells [12]. Consequently, the cell population becomes highly heterogeneous with respect to the cell-specific gene overexpression and thereby also with respect to cell-specific virus yield. The significance of such heterogeneities for the overall production process has been highlighted in prior computational studies [13, 14]. An established method to capture cell-to-cell variability in terms of distributed states and parameters is population balance modeling [14, 15]. Here, the dynamics of intracellular viral components are described by a kinetic model of the viral life cycle [16]. The resulting mathematical model represents a high-dimensional partial-integro-differential equation system for which analytic solutions are not available. However, the complex model equations can be efficiently solved with our recently developed approximate moment method [14].

A direct consideration of the gene variability is a challenging problem and requires a mathematical description of cellular transcription and translation for the corresponding HCFs. Furthermore, interactions of HCFs with the viral life cycle, which itself is still subject of current research [5–9], would have to be taken into account for such detailed description. Alternatively, variability introduced by the gene editing method can be captured by the variance of kinetic parameters. The corresponding parameter distributions are based on bootstrap parameter estimates and are used to account for the aforementioned cellular heterogeneity. Bootstrap parameter estimates were determined on the basis of experimental data sets for SGOs and used to simulate the population balance model of the viral replication process. In a former study, the virus yields of MGOs were analyzed using a random combination of median SGO parameter values in a single cell model [6]. In our contribution, we present a more sophisticated approach, where the random selection of median values is no longer required. To this end, we evaluated four different strategies to generate distributed parameter sets for MGOs based on parameter sets which were estimated from experimental data of their corresponding infected SGOs [6, 17]. In the following, the most suitable strategy was used to generate new parameter distributions for potential MGOs to predict the virus yield in model simulations. Our MGO simulations successfully reproduced the maximum virus titer which is the key characteristic of a vaccine production process. Finally, this combinatorial simulation study allows to predict beneficial gene combinations with respect to the overall virus yield.

In the present paper, cellular heterogeneities were used as additional information in a population balance framework without increasing the model complexity with respect to the number of ordinary differential equations of the internal model for the viral life cycle. Our novel approach allows to generate parameter distributions of MGOs, that were not investigated experimentally, based on data for the associated SGOs. Using these distributions in conjunction with the approximate moment method, it is possible to simulate the virus dynamics of cell lines with new combinations of gene modifications and furthermore predict their efficiency regarding the virus yield in a vaccine production process. Our paper shows one of the few examples in systems biology where high-dimensional population balance models were efficiently solved and used as prediction tool to reduce experimental effort.

## Results and discussion

### Population balance model simulations can capture the trend of the experimental virus yield using different SGOs

**Fig 1:**
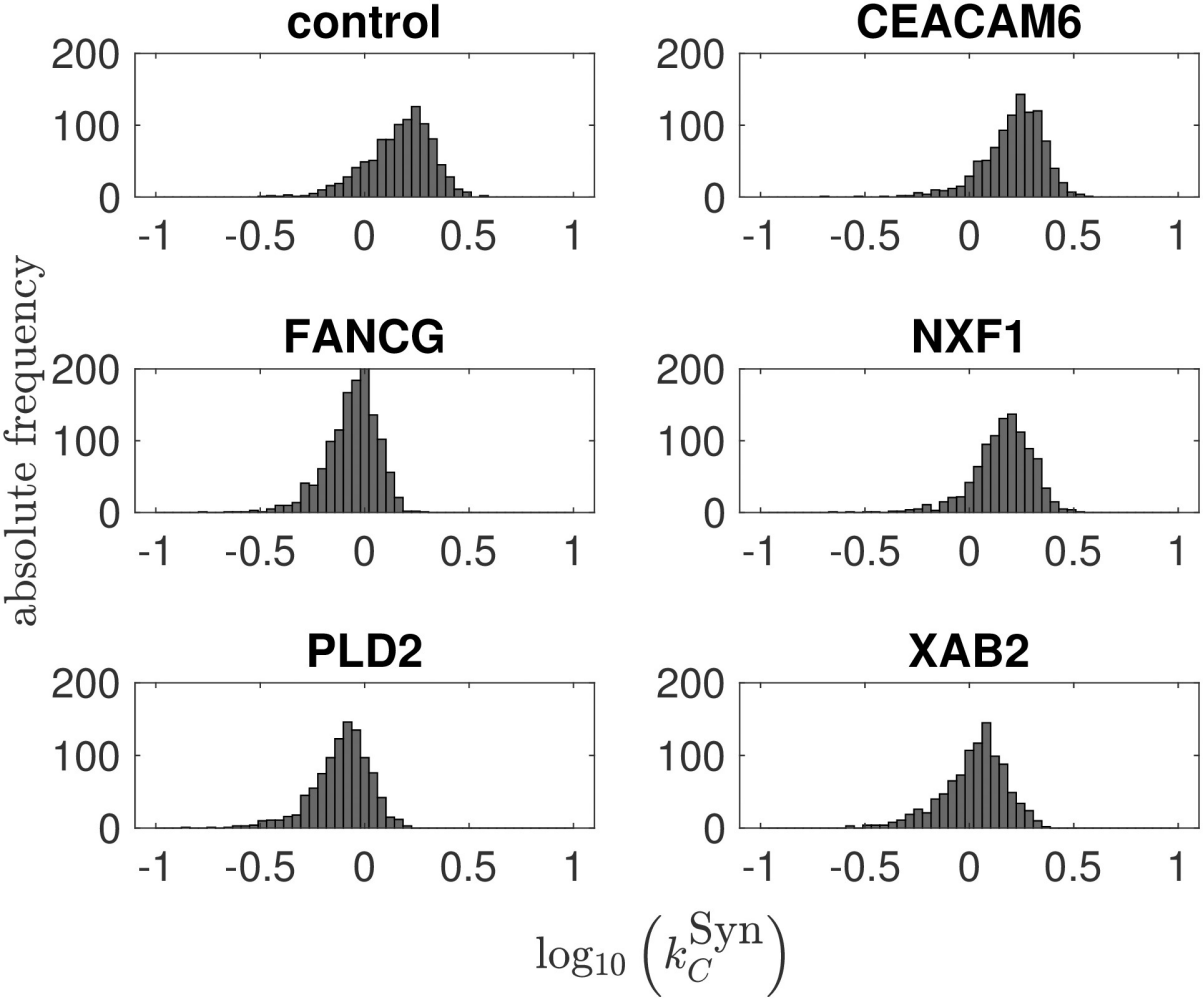
Parameter distributions for the kinetic parameter $log_{10}(k_{C}^{\text{Syn}})$ after a bootstrap parameter estimation with *n* = 1000.

**Fig 2:**
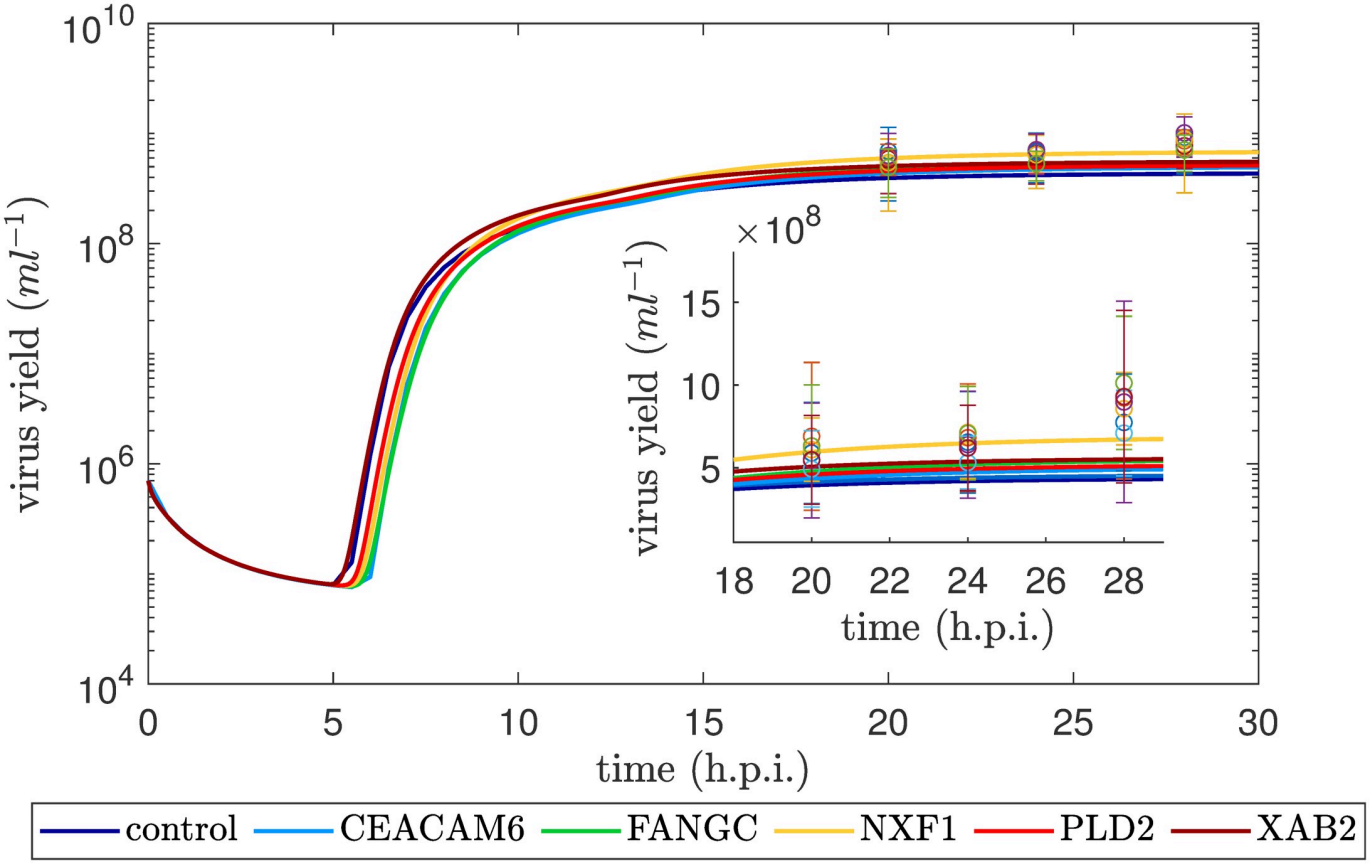
Virus release dynamics for an infection of 7 ⋅ 10^5^ cells at MOI 1. Experimental data are shown as circles with error bars while the simulated values are illustrated as solid lines for five SGOs and the transduction control.

In a first step, experimental data from a previous study on optimization of influenza vaccine production [6] were used to perform a bootstrap parameter estimation with an intracellular deterministic model [16] and an adjusted viral fusion rate (see Materials and methods). The full set of intracellular data [6] comprises flow cytometric measurements of nuclear vRNP import dynamics and the intracellular concentration of viral RNA (mRNA, cRNA, vRNA) of the viral genome segment 5. Furthermore, cell-specific virus yields were measured in the early phase of virus release upon infection of A549 cells at MOI of 1. One exemplary data set for the parental A549 cell line (control) can be found in Fig A in S1 Text. Based on this set of experimental data, parameter distributions were obtained for the synthesis rates of viral mRNA ($k_{M}^{\text{Syn}}$), vRNA ($k_{V}^{\text{Syn}}$), cRNA ($k_{C}^{\text{Syn}}$), the binding rate of M1 to vRNPs ($k_{M1}^{\text{Bind}}$), the release (*k*^Rel^) and import rate of the virus (*k*^Imp^). The resulting distributions for each SGO of the cRNA synthesis rate $k_{C}^{\text{Syn}}$ are shown in Fig 1. The other parameter distributions are illustrated in Fig B–F in S1 Text. As a proof of concept, the population balance model is challenged with the obtained distributions and simulated with 7 ⋅ 10^5^ initial target cells and an MOI of 1 (Fig 2). The usage of the parameter distribution in the population balance framework shows good agreement to the virus titers in the early phase of infection. Though cell age, i.e., the intracellular time span passed since infection, is not directly modeled, an age-like structure, where not all cells are initially infected but some get infected at later stages, emerges from the applied population balance modeling framework. This fact can also be obtained in Fig J in S1 Text. Here, the dynamics of the overall number of uninfected target cells and infected cells is depicted.

### The shift strategy constitutes a suitable approach to construct MGO parameter distributions

**Fig 3:**
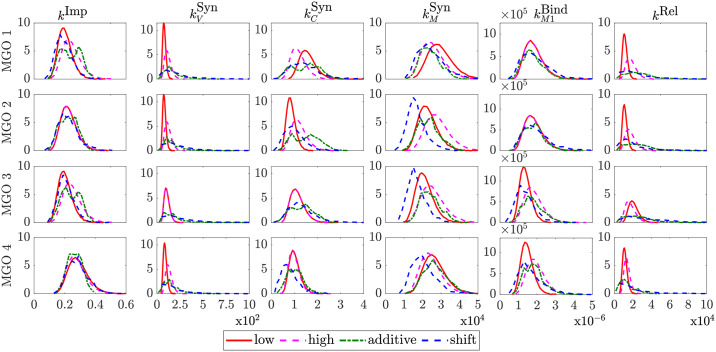
Parameter distributions for MGOs, that were investigated experimentally.

**Fig 4:**
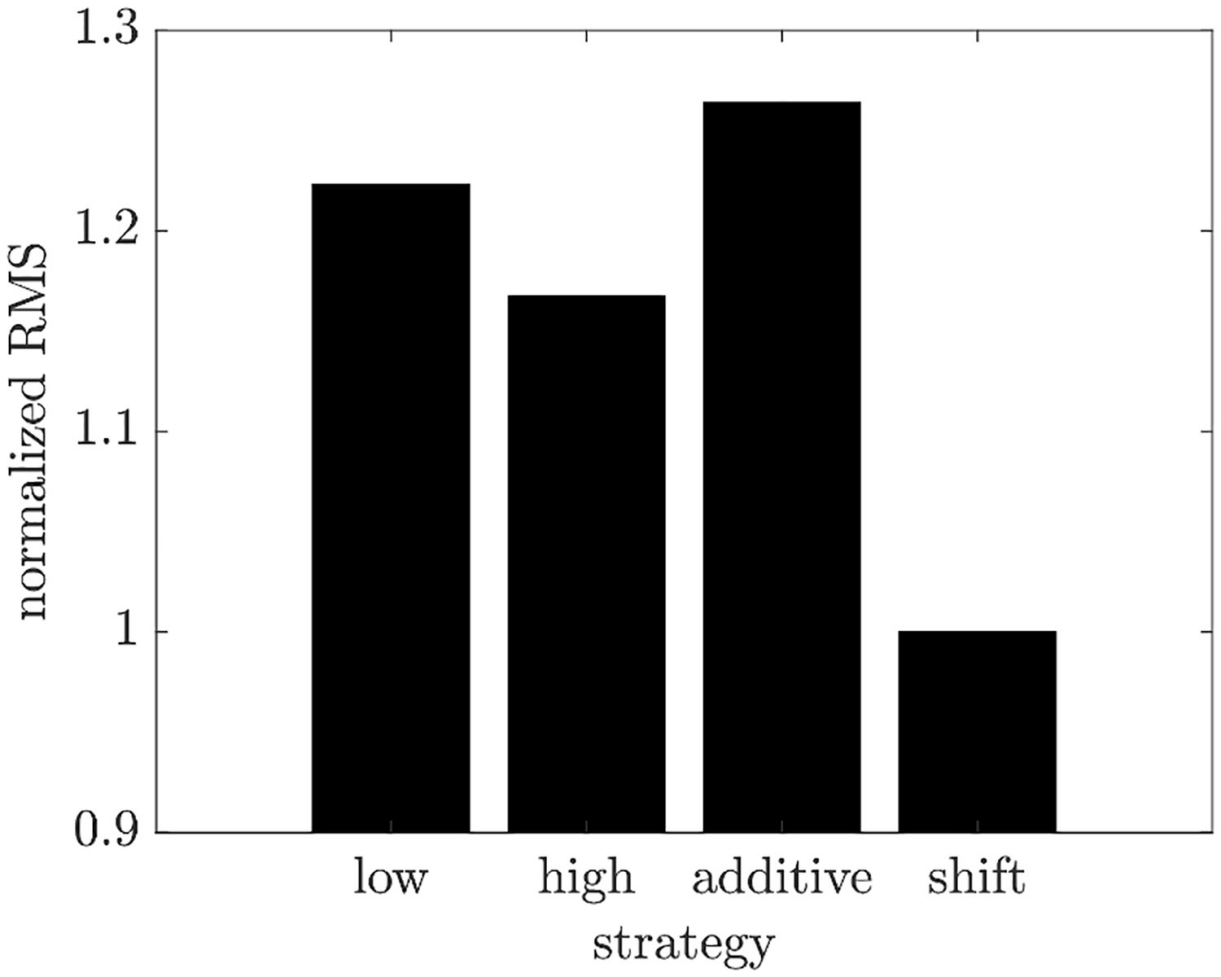
Lumped RMS values for all strategies after comparison of MGO simulations to the experimental data (Fig 5 and Fig H in S1 Text).

**Fig 5:**
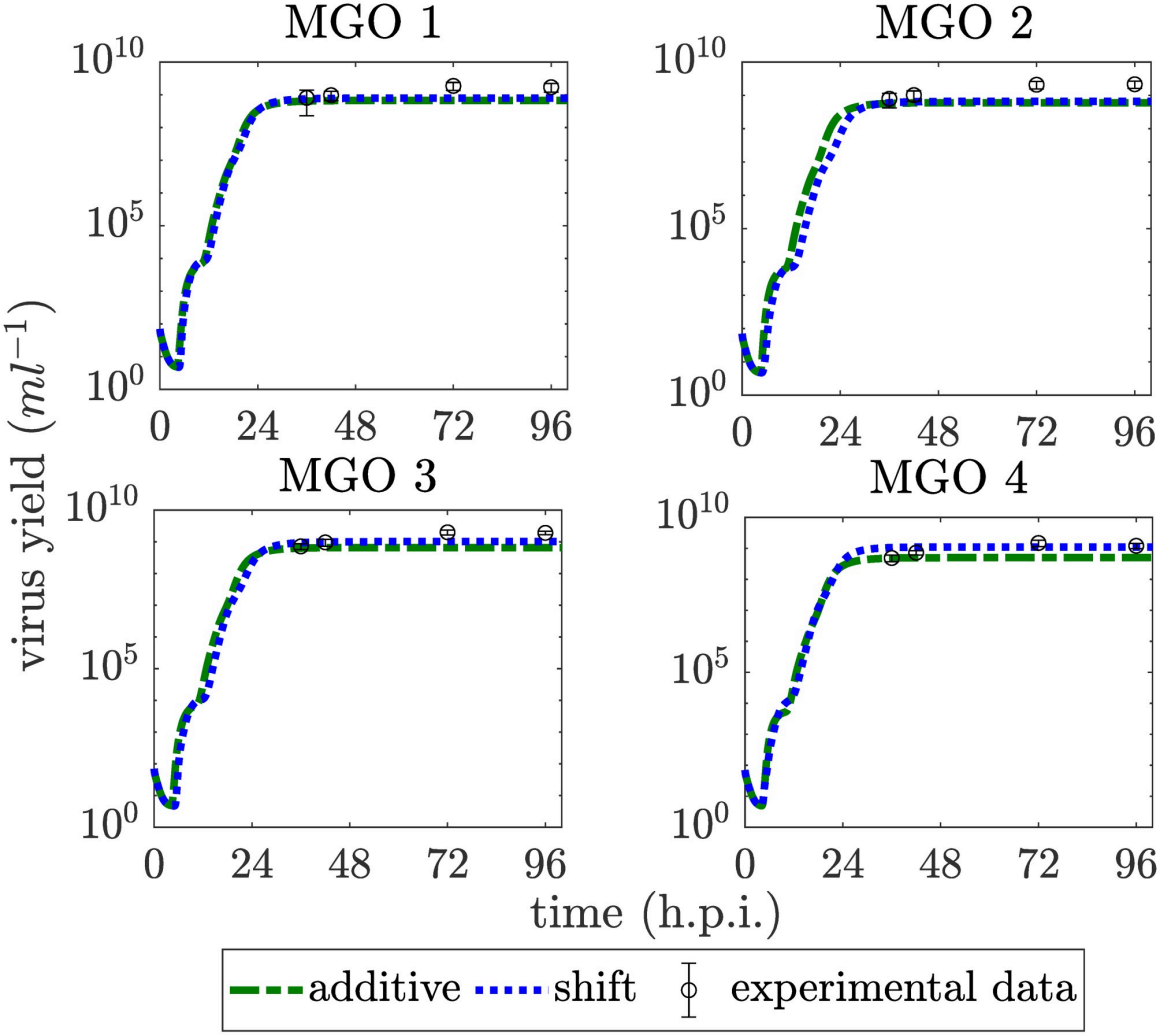
Virus release dynamics for an infection of 7 ⋅ 10^5^ cells at MOI 10^−4^ for the additive and the shift strategy and all MGOs, that were investigated experimentally. Experimental data are shown as circles with error bars while the simulated values are illustrated as green dashed and blue dotted lines.

The observation of the non-significant yield increase for the SGOs in comparison to their control (see Fig 2) further motivated us to investigate MGOs in order to obtain a significant yield increase. For this computational investigation, we generated parameter distributions for MGOs. To validate the strategies for constructing MGO distributions from SGO experiments, the generated parameter distributions (Fig 3) were used to simulate the virus dynamics for the MGOs that were investigated experimentally. Based on the comparison of simulation results with the measured virus concentration and the calculation of the RMS values (Fig 4 and Fig G in S1 Text), the shift strategy shows the best performance for construction of the unknown parameter distributions (see Fig 5 and Fig H in S1 Text). For some of the kinetic parameters, the parameter distributions constructed by the shift strategy follow a gamma-distribution (Fig 3). For instance, the gamma distribution of $k_{V}^{\text{Syn}}$ and *k*^Rel^ results in a cell population in which the majority of cells replicate virus with both a low synthesis of viral RNA and slow viral release dynamics. Only a small proportion of cells propagate virus with high $k_{V}^{\text{Syn}}$ and *k*^Rel^, which may lead to the marginal increase in the virus concentration for the MGOs 1–4 in comparison to SGOs.

### Screening all possible MGO combinations with a model-based approach reduces the experimental effort

**Fig 6:**
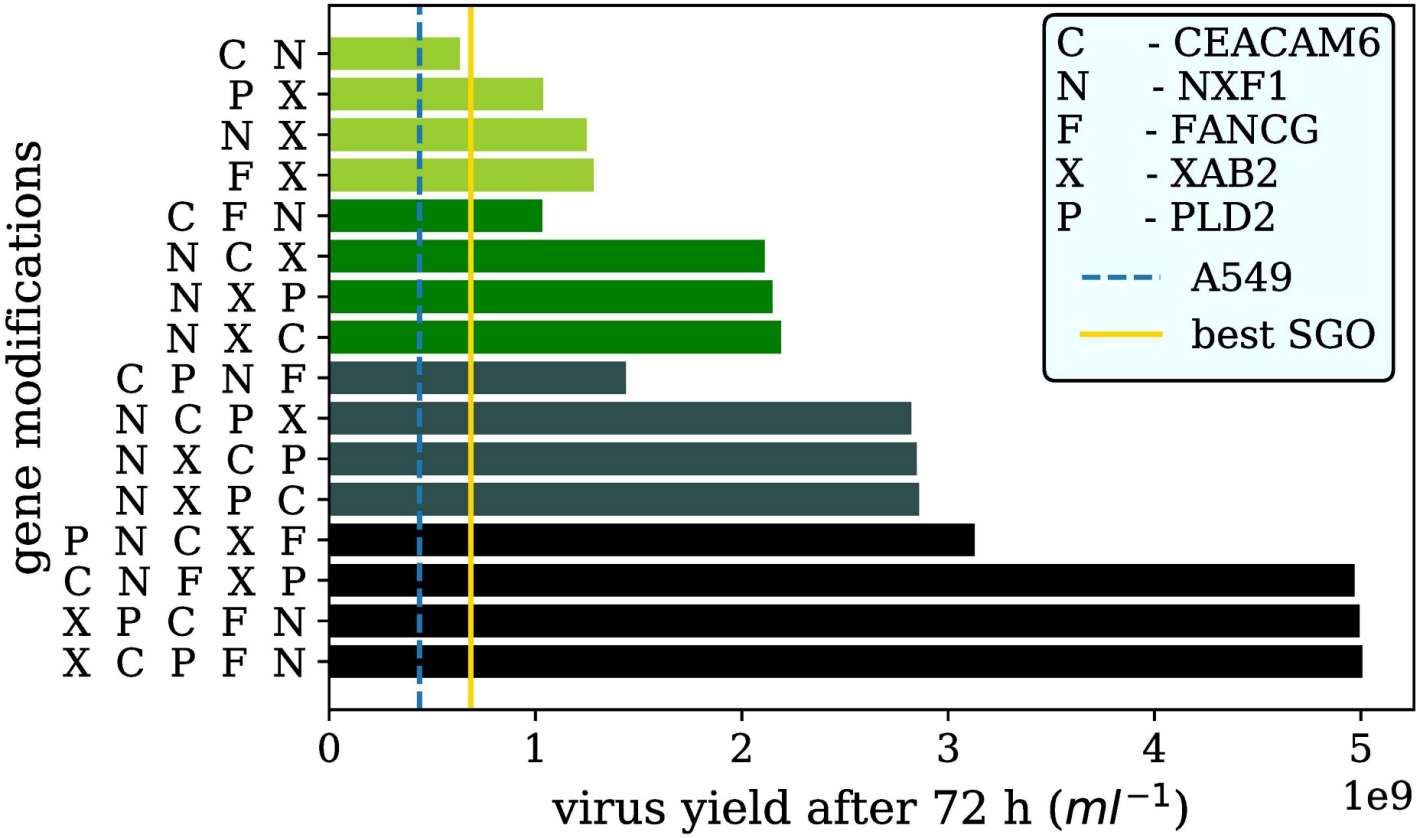
Virus yields after 72 hours of the best three and the least promising combinations using the constructed MGO parameter distributions consisting of two to five SGOs. The coloring of the bars classifies the number of genes modified in the engineered cell lines. The darker the color of the bars the more gene modifications were considered. The order of the gene modifications on the y-axis from left to right corresponds to the order of the stepwise lentiviral transductions made to generate the MGOs. The parental cell line A549 and the best SGO (gene modification in NXF1) were illustrated as dashed blue and solid yellow vertical lines, respectively.

In this section, application of the established shift strategy within a model-based procedure for prediction of promising MGO candidates as alternative to an expensive brute force experimental cell line screening is demonstrated. In total, 320 theoretically possible gene combinations were constructed on the basis of the five known SGOs. Using the resulting parameter distributions, the virus yield at 72 h.p.i. was simulated using the population balance model (Fig 6 and Fig I in S1 Text). In contrast to the evaluation of the shift strategy in the previous section, we focus on the determination of the number and order of gene modifications. In our study the order is important, because the lentiviral transduction, we used here, is a non-targeted gene editing method, but still, it is not a completely random process [33, 34]. While lentiviral gene integration favors loci with a high transcriptional activity, the cellular genome exhibits only a finite number of integration sites [35]. Hence, the more genes are transduced the fewer suitable integration sites are available, i.e., the later a gene construct is transduced during MGO generation, the less likely it is integrated at an active site. Consequentely, an overall decrease in expression level of gene constructs is anticipated during a sequence of multiple transductions applied for MGO engineering. Furthermore, the success of lentiviral transduction also depends on cellular factors, e.g. the nuclear core complex which is responsible for the transportation and perforation of the envelope comprising the gene construct [33]. These effects were considered in our shift strategy by weighting the SGO parameter distributions with the relative fold overexpression according to Eq 20 in the Materials and Methods section. With respect to the order of integrated gene constructs, this means that the production phenotype of a cell line is rather influenced by genes transduced earlier than by those transduced later during MGO engineering. In contrast to the MGOs being investigated experimentally, the selection of only one distribution for the shift of the base parameter distribution is not necessary, because each gene modification is assumed to be included with a separate lentiviral transduction.

Analysis of the three best combinations indicates that the overexpression of NXF1 seems to be a good starting modification for the generation of MGOs (Fig 6). A study from 2014 showed the impact of NXF1 by analyzing influenza intron-less mRNAs after inhibition of NXF1 in A549 cells [9]. The inhibition leads to less hemagglutinin (HA), neuraminidase (NA) and nucleoprotein (NP) mRNAs in the cytoplasm. On the other hand an overexpression of NXF1 could cause an increase of such viral mRNAs. Assuming a robust translation, more viral mRNAs in the cytoplasm result in an increased amount of the viral proteins HA and NA which are the major components of the virus envelope. The increased amount of these viral surface proteins could be a key mechanism for an increased production of virus particles. Besides the enhanced production of HA and NA, NP could be also increased after overexpression of NXF1. NP is part of the vRNP that contains the viral genome. More vRNPs and virus envelope proteins provide improved conditions to overcome the limitation of the virus release, which was determined as kinetic bottleneck by a model-based study of Laske and co-workers [6].

Furthermore, the overexpression of XAB2 in an MGO combination appears to be beneficial for the resulting virus yield (Fig 6). XAB2 is known as an important factor involved in pre-mRNA splicing, cellular transcription and transcription-coupled repair [10]. The nuclear transcription machinery is used by the influenza A virus (IAV), e.g. in case of the 5’-cap snatching from cellular pre-mRNAs. Therefore, it is beneficial for the virus to release the viral genome in a nucleus with an efficient cellular transcription.

An overexpression of XAB2 could enhance these processes. Furthermore, splicing is one of the cellular features that is used to process the mRNAs coding for the ion channel M2 (spliced transcript of the segment 7) or the nuclear export protein (NEP; spliced transcript of the segment 8) [36]. The virus alone is unable to transcribe all necessary proteins without interaction with the cellular splicosome. An up-regulated XAB2 involved in the splicing process might affect the amount of viral transcripts in a positive way and more progeny virus can be released.

The majority of less productive MGOs carried CEACAM6 as base gene modification (Fig 6). This finding is contradictory to the literature [7]: Beside its important role for the release of progeny virus particles, the viral protein NA interacts with CEACAM6 in A549 cells which may promote cell survival during the infection process. Thus, an overexpression of CEACAM6 genes might be a good way to avoid cell death. The effect of an overexpression could, however, be neglected by the fact that an IAV infection elevates the CEACAM6 mRNA and protein level even in unmodified A549 cells [7].

### Conclusion

In this paper, we proposed a new methodology to predict the impact of genetic modifications in face of ubiquitous cellular heterogeneity on the overall yield in biopharmaceutical production processes. Thereby, promising candidates can be determined from computational studies decreasing the number of screening experiments.

Application was demonstrated for influenza vaccine production with cell lines overexpressing certain genes. Here, the overexpression was achieved by lentiviral transduction, a gene editing method for stable integration of gene constructs into the cellular genome. Since lentiviral transduction is a non-targeted method and the number of gene integrations vary within the cell population the cell line becomes highly heterogeneous. Cell-to-cell variability is taken into account in view of intracellular viral components and kinetic parameters affected by the lentiviral transduction within a population balance modeling framework [14, 15].

The parameter distributions for SGOs were determined by bootstrap estimates using experimental data and applied to simulate the virus dynamics for each SGO using population balance modeling. Furthermore, our approach is able to predict the virus dynamics of MGOs on the basis of a few SGO data sets. For this, we evaluated four different strategies to combine SGO parameter distributions in order to obtain the parameter distributions of MGOs. We found that the shift strategy is the most convenient method for this application. We used this strategy together with the population balance model for an *in silico* screening study of possible MGOs that have not yet been investigated experimentally before, and have determined the most promising candidates. The results can be used to support planning of experiments by preselection of gene constructs for combination of genetic modifications to enhance virus yield.

**Fig 7:**
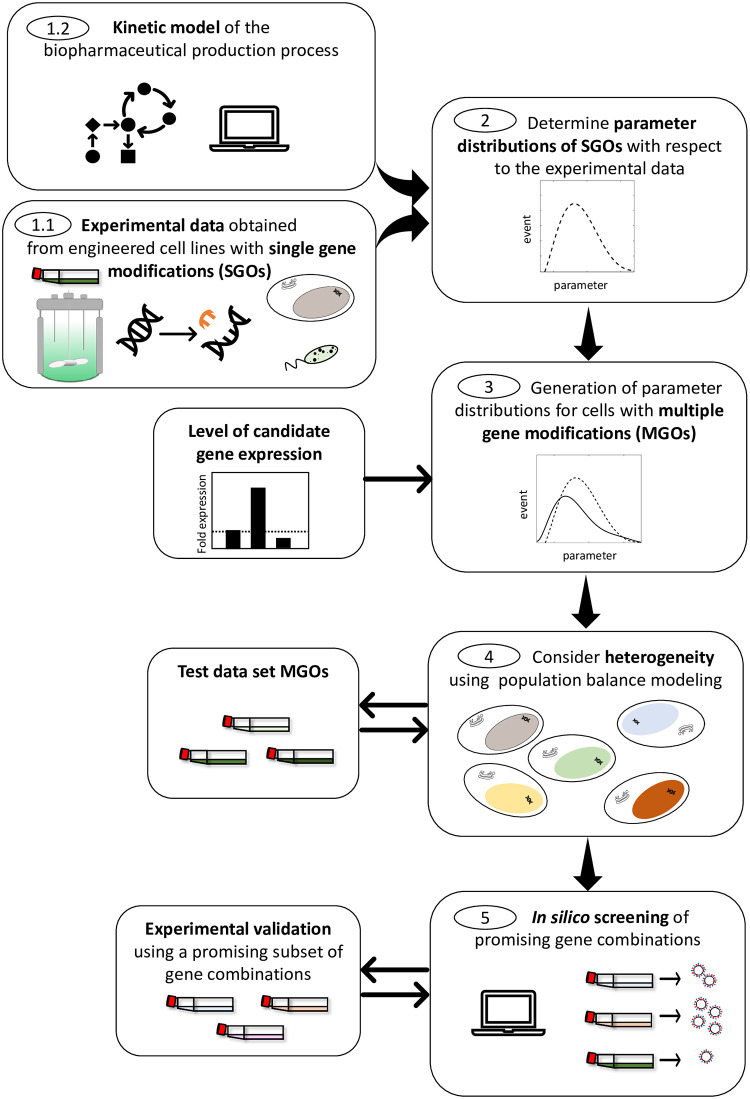
General workflow for the application of the presented methodology on a broad range of biopharmaceutical manufacturing processes.

Here, we presented a proof of concept based on data sets from a study that aimed to improve influenza vaccine production. However, in that study the gene candidates showed only a non-significant impact on final virus yield. Nevertheless, as soon as experimental data from more promising SGOs become available, we could readily apply our method to make predictions on MGOs. The general procedure is summarized in Fig 7. Since we proved our approach to the more complex case of a cytopathic production process further studies could elucidate the impact of our methodology for production processes with non-cytopathic biopharmaceuticals. Thereby, it would be easier to isolate and expand high producer cell lines which might lead to a smaller heterogeneity regarding the gene modification. But even for improved genetic engineering through the control of integration sites, e.g. by the use of the CRISPR/Cas9 [37] or recombinase-mediated cassette exchange method [38], the heterogeneity of cells caused by transduction cannot be completely neglected [39]. In general, natural heterogeneity of the cells leads to different levels of translational and transcriptional activity, which also plays a role in targeted, stable gene integrations. The effect of this natural heterogeneity was shown by Heldt and co-workers, who revealed that the productivity of IAV-infected single cells spans approximately three orders of magnitude [40].

In summary, using suitable and sufficiently complex mathematical models, the influence of heterogeneity on the transcription and translation of gene constructs with multiple gene modifications can be described and, thus, provides a prediction of appropriate combinations for a broad range of biopharmaceutical processes.

## Materials and methods

Experimental methods and setups as well as mathematical modeling and numerical solution techniques are summarized briefly in the following subsections. For further technical details on cell culture maintenance, infection protocols as well as assaying procedures and data analysis the reader is referred to the Methods section in Laske *et al*. (2019) [6]. Further information on population balance modeling of the process and the numerical techniques is found in Dürr *et al*. (2017) [14].

### Lentiviral transduction

Lentiviral transduction was applied to generate A549 cell populations that overexpress host cell genes relevant for IAV replication. We selected CEACAM6, FANCG, NXF1, PLD2 and XAB2 based on RNAi screening [5, 18–20] and various experimental studies [7–9, 21]. The human cDNA sequences of the candidate genes were cloned into the bicistronic lentiviral vector pLV-X-GFPneo, which allowed selection of successfully transduced cells by neomycin resistance. Furthermore, transduced cell populations were subjected to fluorescence activated cell sorting (FACS) to enrich cells that express the transduced gene based on eGFP, which is the co-expressed reporter gene. We used lentiviral transduction without control of the integration site and assumed that cells, for which insertion of the lentiviral constructs was beneficial, will propagate well in culture. MGOs were derived from SGOs by transducing cells with two cocktails containing two to three different lentiviral stocks each on two consecutive days. The resulting MGOs expressed different combinations of the candidate genes at various levels (Table 1). For further details on generation and production of lentiviral vectors, and the transduction procedure the reader is referred to the Methods section of Laske *et al*. (2019) [6].

**Table 1:** Gene combinations in MGOs. The checks mark the candidate gene that was overexpressed first. Grey shading indicates further overexpressed genes in the corresponding MGOs.

	CEACAM6	XAB2	FANCG	PLD2	NXF1
MGO 1	√				
MGO 2			√		
MGO 3					√
MGO 4		√			

### Infection experiments

#### Cell culture and virus infection

For the IAV infection, A549 cell lines were seeded into multiple 12-well plates and incubated over night at 37°C and 5% CO_2_ atmosphere. On the next day, cells reached a cell number of approximately 7 ⋅ 10^5^ cells per well and were infected with the influenza virus strain A/Puerto Rico/8/34 (A/PR/8/34, H1N1). To synchronize infection and facilitate parameter inference for intracellular IAV replication and virus release, cells were infected at multiplicity of infection (MOI) 50 and MOI 1, respectively (see Fig A in S1 Text). Multiple cycle progression of IAV infection was investigated in A549 cells infected at MOI 10^−4^.

#### Imaging flow cytometry

For the flow cytometric measurement of nuclear vRNP (viral ribonucleoprotein) import, A549 cells were treated with the translation inhibitor cycloheximide (CHX) prior and during infection at MOI 50. Infected cells were harvested at multiple time points post infection, they were fixated and co-stained by DAPI, a nuclear dye, and the mAb64A5 antibody [22] that preferentially binds oligomerized viral nucleoprotein (NP) which is present predominantly in the IAV RNP complex. Using imaging flow cytometry (ImageStream X Mark II, Amnis, EMD Millipore) the relative intensity of the vRNP signal inside the nucleus was measured every 15 minutes for 2 hours post infection (h.p.i., see Fig A in S1 Text).

#### Real-time RT-qPCR

The intracellular viral RNA copy numbers of segment 5 (encoding viral NP) were quantified by real-time RT-qPCR from lysates of cells infected at MOI 50 (see Fig A in S1 Text). In particular, the three viral RNA species (mRNA, cRNA, vRNA) were distinguished using polarity- and gene-specific tagged primers (as detailed in [23]).

The $2^{-\Delta \Delta C_{T}}$ method [24] was used to calculate the relative overexpression level of candidate genes in SGOs compared to the parental A549 cell lines using the 18S rRNA as a calibrator.

#### Virus quantification

Virus titers were determined by hemagglutination assay (HA assay) [25]. Based on the measurement result of the HA assay in log_10_HA units per test volume (*log*_10_ *HAU*/100*μL*) and the cell concentration of the erythrocyte suspension (2 ⋅ 10^7^ cells/mL), the total virus particle concentration can be calculated as follows:

$$\begin{array}{c} c_{virus}=2\cdot 10^{7}\cdot 10^{\left(log_{10}HAU/100\mu L\right)}\text{.} \end{array}$$ (1)

In the infection experiment with an MOI of 1 the HA assay was performed for samples collected at 20, 24 and 28 h.p.i. (Fig 2 and Fig A in S1 Text).

### Mathematical model

**Fig 8:**
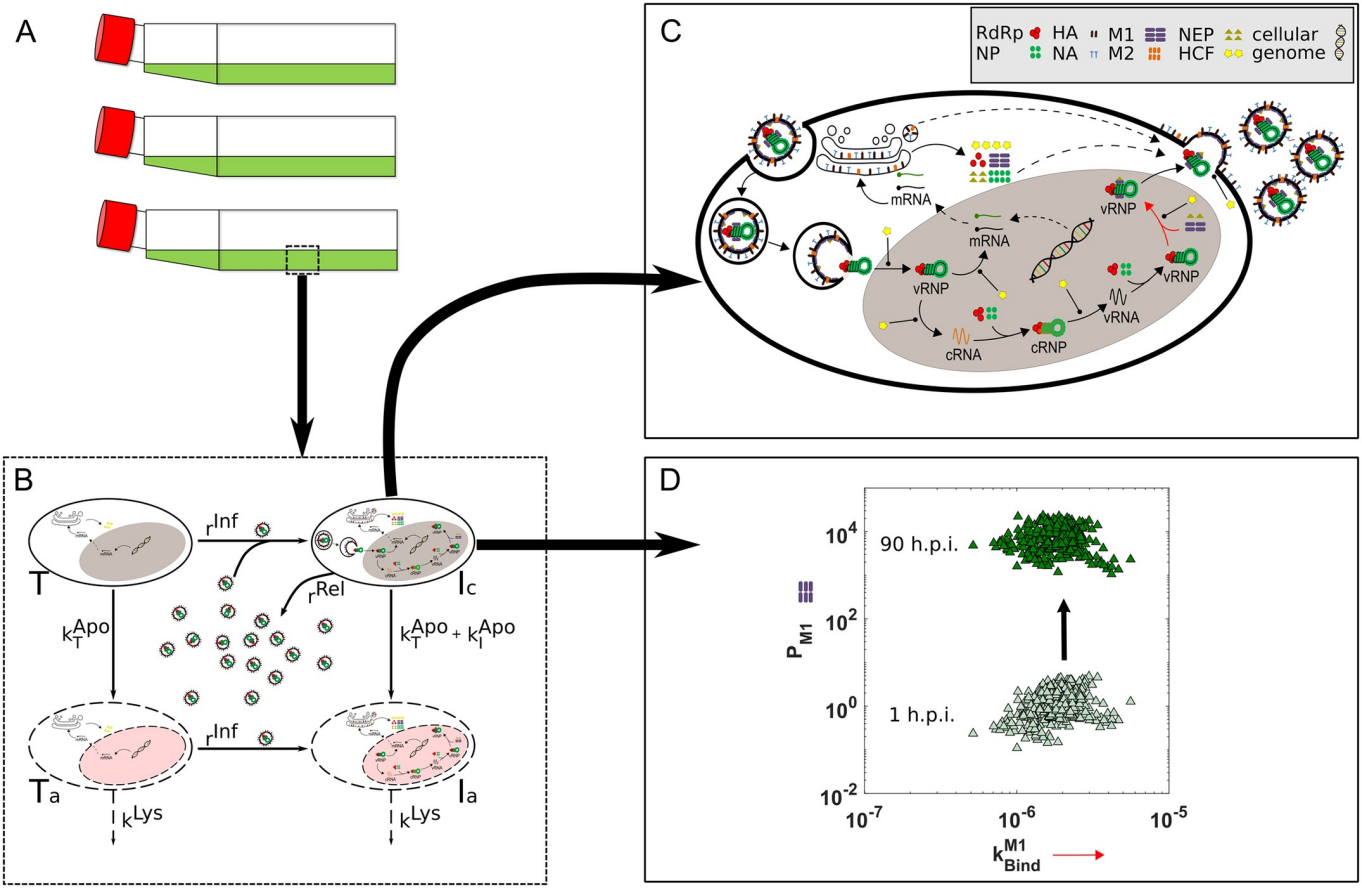
Mathematical model with different scales based on [14, 16]. Production of virus particles in mammalian cells can be investigated in T-flask experiments (A). The extracellular model is represented by the dashed box (B). Uninfected target cells can be infected by virus particles. Both infected and uninfected cells can become apoptotic. The intracellular model describes the replication of the viral genome (C). The mathematical description of the intracellular model starts with the virus in the endosome. Different intracellular stages can be influenced by several HCFs. After transcription and translation of the viral genome new virus particles can be released to the surrounding medium. The rates affected by genetic modification (arrows in C) are marked with the yellow asterisks. As an example, the distribution of the matrix protein M1 (*P*_*M*1_) and the parameter $k_{M1}^{\text{Bind}}$ (red arrow in C) are shown for two different time points (D).

Population balance modeling [26] is an established framework to account for heterogeneity in multicellular systems and can thus be applied to describe the observed cell-to-cell variability with respect to the intracellular viral components and the kinetic parameters affected by the genetic modifications. The resulting model describes the virus production process on multiple scales which account for intra- and extracellular dynamics (see Fig 8): The intracellular level accounts for the major steps of the viral life cycle with focus on RNA replication and regulation (see [16] for detailed information on the intracellular viral replication kinetics) while the extracellular level characterizes interaction of cell and virus species. In the following, we will summarize the resulting multiscale model formulation as presented in [13]:

We assume that cell-to-cell variability of infected cells can be considered by distributed intracellular viral components and distributed kinetic parameters. These result in distributed dynamics of the cell population, i.e., individual cells express different behaviors. In Fig 8D an example for the temporal evolution of the number density of cells depending on the distributed intracellular state *P*_*M*1_ and the distributed kinetic parameter $k_{M1}^{\text{Bind}}$ can be seen. The dynamics of the heterogeneous cell population is characterized by the temporal evolution of the cell number density distribution *i*_*c*_(*t*, **x**) which follows from the multidimensional population balance equation:

$$\begin{array}{c} \frac{\partial i_{c}}{\partial t}+\nabla_{x^{*}}(h^{*}\;i_{c})=-\left(k_{T}^{\text{Apo}}+k_{I}^{\text{Apo}}\right)i_{c}+r^{\text{Inf}}\;T\;I(x)\;. \end{array}$$ (2)

Here, the extended single cell dynamics h* = [**h**, **0**]^*T*^ of the extended state vector x* = [**x**, **k**]^*T*^ describes the temporal evolution of the number density of cells depending on the different intracellular states **x** (containing for instance mRNA species and viral proteins) and the kinetic parameters **k**. The single cell dynamics are given correspondingly by **h** and described in more detail in [14]. In this study, we assume, that cell-to-cell variability resulting from lentiviral transduction translates directly to distributed kinetic parameters represented by the vector **k** which vary within the population of infected cells and are time invariant. We further assume, that only the rate of nuclear vRNP import, the synthesis rates of viral mRNA, cRNA, vRNA, the binding rate of M1 to nuclear vRNPs, and the release rate of virus particles are affected, thus

$$\begin{array}{c} k=[\;k^{\text{Imp}},\;k_{M}^{\text{Syn}},\;k_{C}^{\text{Syn}},\;k_{V}^{\text{Syn}},\;k_{M1}^{\text{Bind}},\;k^{\text{Rel}}]. \end{array}$$ (3)

The number of infected cells increases by infection of uninfected cells with rate *r*^inf^. Furthermore, the distribution I(x) represents the normalized initial cell-to-cell variability of newly infected cells. The population of infected cells decreases due to apoptosis. Here, the apoptotic rate is divided into an apoptotic rate caused by infection $k_{I}^{\text{Apo}}$ and a natural apoptosis rate $k_{T}^{\text{Apo}}$.

The infected cell dynamics is coupled to the temporal evolution of apoptotic cells *I*_*a*_

$$\begin{array}{c} \frac{\text{d}I_{a}}{\text{d}t}=\int_{\text{X}}(k_{T}^{\text{Apo}}+k_{I}^{\text{Apo}})i_{c}\;\text{d}\text{x}+r^{\text{Inf}}\;T_{a}-k^{\text{Lys}}\;I_{a}, \end{array}$$ (4)

viable target cells *T*

$$\begin{array}{cc} \frac{\text{d}T}{\text{d}t} & =g\;T-r^{\text{Inf}}\;T-k_{T}^{\text{Apo}}\;T, \end{array}$$ (5)

and apoptotic target cells *T*_*a*_

$$\begin{array}{cc} \frac{\text{d}T_{a}}{\text{d}t} & =k_{T}^{\text{Apo}}\;T-r^{\text{Inf}}\;T_{a}-k^{\text{Lys}}\;T_{a}, \end{array}$$ (6)

and virus particles *V* as described below. In contrast to infected cells, these species are assumed to be non-distributed, i.e., those species are concentrated. The growth rate *g* is defined as

$$\begin{array}{c} g=[\frac{g_{\text{max}}}{T_{\text{max}}}(T_{\text{max}}-T-\int_{\text{X}}i_{c}\;\text{d}\text{x})]. \end{array}$$ (7)

Virus particles were distinguished depending on their location. There are free active virus particles *V* located in the extracellular medium, virus particles attached to the surface of target cells *V*^Att^ and virions located in endosomes of infected target cells *V*^En^. Their dynamics read as:

$$\begin{array}{cc} \frac{\text{d}V}{\text{d}t} & =\int_{\text{X}}r^{\text{Rel}}\;i_{c}\;\text{d}\text{x}-k_{V}^{\text{Deg}}\;V+\sum_{n}[k_{n}^{\text{Dis}}\;V_{n}^{\text{Att}}-k_{c,n}^{\text{Att}}B_{n}\;V], \end{array}$$ (8)

$$\begin{array}{c} \frac{\text{d}V_{n}^{\text{Att}}}{\text{d}t}=k_{c,n}^{\text{Att}}\;B_{n}\;V-(k_{n}^{\text{Dis}}+k^{\text{En}})\;V_{n}^{\text{Att}}-(r^{\text{Inf}}+r^{\text{Lys}})\;V_{n}^{\text{Att}}, \end{array}$$ (9)

$$\begin{array}{c} \frac{\text{d}V^{\text{En}}}{\text{d}t}=k^{\text{En}}(V_{hi}^{\text{Att}}+V_{lo}^{\text{Att}})-k^{\text{Fus}}\;V^{\text{En}}-(r^{\text{Inf}}+r^{\text{Lys}})\;V^{\text{En}}, \end{array}$$ (10)

with

$$\begin{array}{c} B_{n}=B_{n}^{\text{tot}}(T+T_{a})-V_{n}^{\text{Att}}\;k_{n}^{\text{Dis}}=\frac{k_{c,n}^{\text{Att}}}{k_{c,n}^{\text{Equ}}}\;n\in \{lo,\;hi\}. \end{array}$$ (11)

Two types of binding sites for the virus particles on the surface are considered: low affinity (*lo*) and high affinity (*hi*). A detailed description of the involved kinetic processes can be found in [27]. The infection and cell lysis rates are defined as:

$$\begin{array}{c} r^{\text{inf}}=\frac{F_{\text{inf}}k^{\text{Fus}}V^{\text{En}}}{T+T_{a}}\;,\;r^{\text{lys}}=\frac{k^{\text{Lys}}T_{a}}{T+T_{a}} \end{array}$$ (12)

The viral release rate depends on the amounts of viral components in the cells and is given by

$$\begin{array}{c} r^{\text{Rel}}\left(x\right)=k^{\text{Rel}}\frac{Vp_{M1}^{\text{cyt}}}{Vp_{M1}^{\text{cyt}}+8\;K_{V_{rel}}}\prod_{j}\frac{P_{j}}{P_{j}+N_{j}\;K_{Vrel}}\;,\\ j\;\in \;\{RdRp,\;P_{HA},\;P_{NP},\;P_{NA},\;P_{M1},\;P_{M2},\;P_{NEP}\}. \end{array}$$ (13)

All kinetic model parameters can be found in Table 2. Here, virus entry is considered on the macroscopic scale and newly infected cells are initialized with a complete set of eight vRNP segments.

**Table 2:** Kinetic model parameters for the extracellular model equations.

Parameter	Value	Unit	Parameter	Value	Unit
$B_{hi}^{\text{tot}}$	150	sites/cell	*k*^Rel^	586 virions/h	
$B_{lo}^{\text{tot}}$	1000	sites/cell	$k_{T}^{\text{Apo}}$	7.35*10^−3^	h^−1^
*F*_inf_	1	cells/virions	$k_{V}^{\text{Deg}}$	0.1	h^−1^
*g*_*max*_	0.03	h^−1^	*K*_*Vrel*_	300	virions
$k_{c,hi}^{\text{Att}}$	3.32*10^−8^	mL/(sites h)	*T*_max_	7*10^5^	cells/mL
$k_{c,lo}^{\text{Att}}$	1.85*10^−10^	mL/(sites h)	$N_{P_{HA}}$	500	molecules/virion
$k_{c,hi}^{\text{Equ}}$	4.48*10^−9^	mL/site	$N_{P_{NA}}$	100	molecules/virion
$k_{c,lo}^{\text{Equ}}$	3.32*10^−11^	mL/site	$N_{P_{NEP}}$	165	molecules/virion
*k*^En^	4.8	h^−1^	$N_{P_{NP}}$	1000	molecules/virion
*k*^Fus^	3.21	h^−1^	$N_{P_{M1}}$	3000	molecules/virion
$k_{I}^{\text{Apo}}$	3.28*10^−2^	h^−1^	$N_{P_{M2}}$	40	molecules/virion
*k*^Lys^	6.39*10^−2^	h^−1^	*N*_*RdRp*_	45	molecules/virion

### Estimation of distributed parameters from SGO infection experiments

To obtain parameter distributions for the affected kinetic parameters **k** of the population balance model, the parameter set of the intracellular model for IAV replication was re-calibrated to experimental data obtained from infected engineered cell lines as recently published by Laske *et al*. (2019) [6]. In contrast to [6], we used the intracellular model as published in [16] with the single modification that the fusion rate of virions in late endosomes is *k*^Fus^ = 3.21 *h*^−1^.

Given the available experimental data, we determined the parameter distributions of the nuclear import rate of vRNPs *k*^*Imp*^, the synthesis rates of the three viral RNA species ($k_{M}^{\text{Syn}},\;k_{C}^{\text{Syn}},\;k_{V}^{\text{Syn}}$), the binding rate of the matrix protein 1 (M1) $k_{M1}^{\text{Bind}}$ to nuclear vRNPs as well as the release rate of progeny virions *k*^Rel^, which were selected as distributed parameter vector, see Eq 3.

Estimation of parameter distributions was performed in two consecutive steps. First, nuclear vRNP import was assessed from infected cells treated with the translation inhibitor CHX (see Fig A in S1 Text). Due to the inhibition of protein synthesis no viral proteins are produced and therefore, viral genomes cannot replicate. Consequently, only the incoming vRNPs are stained by the anti-vRNP antibody and their nuclear import rate is estimated by fitting the simulated fraction of nuclear vRNPs $frac_{\text{Rnp}}^{\text{nuc}}$ to the averaged relative fluorescence intensity of the nucleus $frac_{\text{Int}}^{\text{nuc}}$.

$$\begin{array}{c} Rnp^{\text{cyt}}=8\cdot V^{\text{En}}+Vp^{\text{cyt}}+Vp_{M1}^{\text{cyt}}\text{,} \end{array}$$ (14)

$$\begin{array}{c} Rnp^{\text{nuc}}=Vp^{\text{nuc}}+Vp_{M1}^{\text{nuc}}\text{,} \end{array}$$ (15)

$$\begin{array}{c} frac_{\text{Rnp}}^{\text{nuc}}=(\frac{Rnp^{\text{nuc}}}{Rnp^{\text{nuc}}+Rnp^{\text{cyt}}})\cdot 100\text{.} \end{array}$$ (16)

As can be seen in Eq 14 the vRNPs in the cytoplasm *Rnp*^cyt^ are the sum of the eight vRNPs of virions in early endosomes *V*^En^, vRNPs in the cytoplasm *Vp*^cyt^ and the M1-vRNP complexes in the cytoplasm $Vp_{M1}^{\text{cyt}}$. Furthermore, the amount of nuclear vRNPs *Rnp*^nuc^ is the sum of vRNPs *Vp*^nuc^ and M1-vRNP complexes $Vp_{M1}^{\text{nuc}}$ inside the nucleus (Eq 15).

To estimate the nuclear import rate of vRNPs *k*^Imp^, the square error

$$\begin{array}{c} J=\sum_{k=0}^{T}{\left(\frac{frac_{\text{Rnp}}^{\text{nuc}}\left(t_{k}\right)-frac_{\text{Int}}^{\text{nuc}}\left(t_{k}\right)}{max(frac_{\text{Int}}^{\text{nuc}})}\right)}^{2}\text{,} \end{array}$$ (17)

is minimized. Therein, the distance between the relative fluorescence intensity of the nucleus $frac_{\text{Int}}^{\text{nuc}}$ and the simulated fraction of nuclear vRNPs $frac_{\text{Rnp}}^{\text{nuc}}$ is weighted by the maximal experimental value $max(frac_{\text{Int}}^{\text{nuc}})$.

In a second step, the optimized import rate is kept fixed while the remaining parameter distributions of the model parameter vector (see Eq 3) are estimated from experimental data monitoring intracellular viral RNA transcription and replication by real-time RT-qPCR as well as the release of progeny virions by HA assay over time (see Fig A in S1 Text). Parameter inference was performed by minimizing the squared errors based on the common logarithm of the simulated state values and experimental data as follows:

$$\begin{array}{c} J^{\text{log}}=\sum_{i=1}^{n}\sum_{k=0}^{T}{\left(\frac{log_{10}\left({\text{prediction}}_{i}\left(t_{k}\right)\right)-log_{10}\left({\text{data}}_{i}\left(t_{k}\right)\right)}{\text{max}(log_{10}\left({\text{data}}_{i}\right))}\right)}^{2}. \end{array}$$ (18)

The error of each *i*-th state *n* is weighted by the corresponding maximal logarithmic experimental value max(*log*_10_(data_*i*_)).

Parameter distributions were determined by parametric bootstrapping (details see [28]) using the global stochastic optimization algorithm fSSm [29]. For this, multiple optimization runs were performed to fit 1000 randomly resampled measurement points with respect to the data’s mean and standard deviation. The number of resamples guarantes convergence of the average and standard deviation of the bootstrapped parameter values. The obtained bootstrap parameter distributions are used in the present population balance model to characterize the heterogeneity of virus replication steps in the cell population.

### Numerical solution of the population balance model

The population balance equation (Eq 2) is a multi-dimensional partial-integro-differential equation which is coupled to a set of ordinary differential equations (Eqs 4–10). In general, the dimension of the partial differential equation corresponds to the dimension of the extended state vector x* which comprises the intracellular viral components (*dim*(**x**) = 27) and the distributed kinetic parameters (*dim*(**k**) = 6). Since standard numerical methods for full solution suffer from an enormous numerical effort, we used our recently developed approximate moment method for a numerical solution [14, 30]. This efficient technique combines the direct quadrature method of moments [31] with an efficient choice of quadrature abscissas based on monomial cubatures (see e.g. [32]). In contrast to classical discretization-based methods, the technique relies on the solution of a relatively small number of ordinary differential equations (ODEs) which are used to approximate integral quantities of the full cell number density distribution like mean values and variances with respect to the intracellular states. Thereby, the numerical effort can be reduced significantly: The approximate moment method scales polynomially (at best even linearly) with dimension of the extended state vector x* in contrast to discretization-based approaches, which scale exponentially. More details on the technique are found in [14]. The numerical technique was coded in MATLAB 2016b and the routine *ode15s* was used for solution of the resulting ODE systems.

### Parameter distribution strategies for MGOs and selection criterion

To avoid extensive experimental screening, mathematical modeling can be used to predict virus yields of MGOs. In a previous study by Laske and co-workers [6] median values of the parameter distributions were combined randomly for the six parameters (see Eq 3) and the new parameter sets were used to simulate the virus dynamics using a single cell model as presented in [27]. In contrast, we apply a more sophisticated approach, where MGO parameter distributions are generated based on the entire parameter distributions of their underlying SGOs. Finding a suitable strategy for the construction of MGO parameter distributions was one goal of this investigation. The four strategies tested were programmed in MATLAB 2016b and the resulting parameter distributions were used to simulate the virus dynamics using a population balance modeling approach. The latter were validated against measured virus concentrations from selected MGOs (see Table 1).

#### Low impact strategy

The first strategy is based on the assumption, that the first gene overexpression has the strongest impact while further modifications have only a low impact on the viral life cycle. Thus, the new parameter distributions are located between the median values of the first SGO (base) and the median values of the SGO with the smallest distance to the base median value. The parameter distribution are generated by combining five logarithmic Gaussians in a weighted sum:

$$\begin{array}{c} k_{i}∼\sum_{l=1}^{5}a_{l}\;\mu_{l}\;e^{N(0,\sigma_{l})}. \end{array}$$ (19)

**Fig 9:**
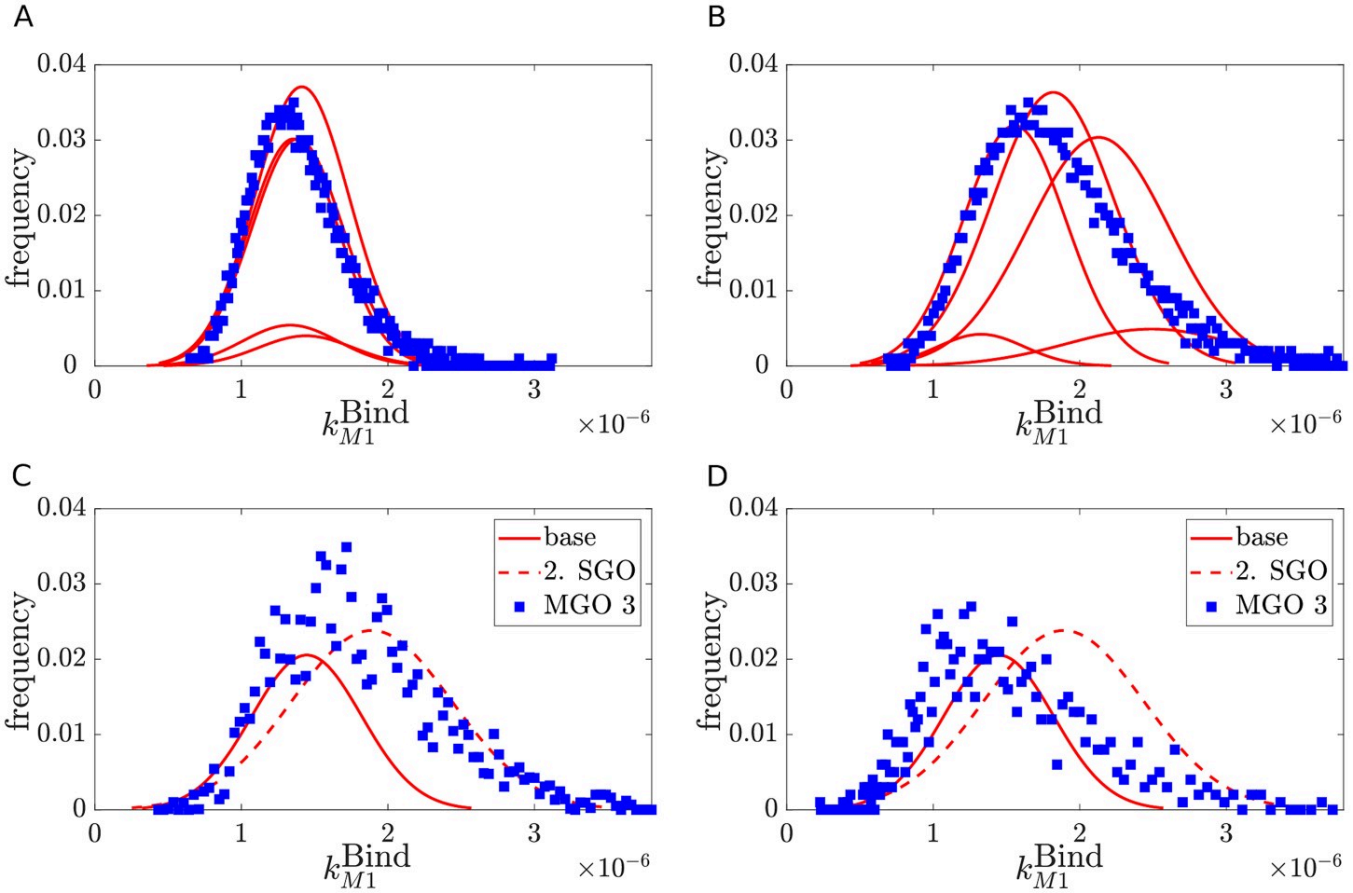
Distribution of the parameter $k_{Bind}^{M1}$ in MGO 3 (blue rectangles) for the low impact (A), high impact (B), additive (C) and shift strategy (D). For the low and high impact strategy the distributions were assembled with five Gaussians (red curves). The position of the Gaussians were obtained as described in the corresponding subsections. For the additive and shift strategy, bootstrap parameter estimates (solid and dashed red curves) were used to generate the distribution of the MGO (blue rectangles). Distributions are normalized with respect to the total number of infected cells.

A previous study discussed the impact of differently shaped parameter distributions [13]. The shape of a parameter distribution can be influenced by the scaling parameter *a*_*l*_. Here, we select a broad final distribution as realistic scenario for a parameter distribution after lentiviral transduction. The corresponding scaling factors are: [*a*_1_, *a*_2_, *a*_3_, *a*_4_, *a*_5_] = [0.05, 0.3, 0.3, 0.3, 0.05]. A representative distribution for the parameter $k_{Bind}^{M1}$ is shown in Fig 9A.

#### High impact strategy

This method for generation of the parameter distributions is similar to the low impact strategy. In comparison to the previous method, we assume that further gene modifications have an equally high impact. For that reason, parameters of the MGOs are distributed between the median values of the base and the median values of the SGO which cause the greatest change in the parameter distribution. Thus, the parameter distributions are broader than in the low impact strategy (Fig 9B).

#### Additive strategy

For the additive strategy, parameter distributions estimated by bootstrapping of experimental data obtained from SGOs are applied. The generation of 150 bins between the minimum and maximum value for each parameter facilitates the comparison of the base parameter distributions with the parameter distributions achieved after a second gene modification. The second set of parameter distributions were selected as in the high impact strategy regarding their maximum absolute distance to the median values of the base SGO. The number of cells with the same parameter value as the corresponding bin was counted for each of them and the resulting distributions were interpolated linearly by using the MATLAB command *interp1* (default setting). To obtain the MGO parameter distributions, an overlay of the base SGO parameter distribution and the second SGO parameter distribution is conducted to select the highest absolute frequency for the six parameters (Fig 9C).

#### Shift strategy

In the shift strategy, parameter distributions obtained by bootstrapping were applied together with the fold overexpression level (FOE) measured by RT-qPCR (see Table A in S1 Text) to generate MGO parameter distributions. In comparison to the previous strategy, we determined mixed Gaussian distributions by using the logarithmic bootstrap parameter estimates from each SGO and the MATLAB function *fitgmdist*. For the validation with the measured virus concentrations of MGO 1-4 (Table 1, Fig 5) a selection of the second parameter distribution is required to perform the shift. As before, the maximum absolute distance to the median values of the base SGO is the selection criterion for the second set of SGO parameter distributions.

To correlate the FOEs, the relative frequency *EL*_*i*,*Rel*_ for each SGO is calculated as follows:

$$\begin{array}{cc} EL_{i,\text{Rel}} & =\frac{FOE_{i}}{\sum_{i}FOE_{i}}\;,\\ \text{with}\;\text{i} & =\{\text{CEACAM6},\;\text{XAB2},\;\text{PLD2},\;\text{NXF1},\;\text{FANCG}\}. \end{array}$$ (20)

The new mixed Gaussian distributions for the MGOs were constructed by using the MATLAB command *gmdistribution*. For that purpose, the relative distance Δ_*i*,*Rel*_ was calculated by multiplication of the relative expression level *EL*_*i*,*Rel*_ and distance of the median value of the base and the second SGO parameter distribution Δ_*i*_:

$$\begin{array}{cc} \Delta_{i,\text{Rel}} & =EL_{i,\text{Rel}}\cdot \Delta_{i}\;,\\ \text{with}\;\text{i} & =\{\text{CEACAM6},\;\text{XAB2},\;\text{PLD2},\;\text{NXF1},\;\text{FANCG}\}. \end{array}$$ (21)

To shift the parameter distributions of the base, a summation of the 1000 bootstrap estimates of the base with the relative distance of the median values of the base and the second SGO was done (Fig 9D). The covariance of each element of the new mixed Gaussian distribution is the standard deviation of the second parameter distribution applied for the shift of the base parameter distribution.

In the subsequent combinatorial study, MGOs were generated *in silico* by assuming that each gene overexpression is achieved in an individual lentiviral transduction event. Therefore, the shift of the distribution for each parameter was done stepwise considering the order of gene modifications in the MGO.

#### Strategy selection

The root mean square (RMS) error

$$\begin{array}{c} RMS=\sqrt{\frac{1}{m}\sum_{i=1}^{m}{\left(log\left(y_{i}\right)-log\left(\bar{y_{i}}\right)\right)}^{2}} \end{array}$$ (22)

is used to evaluate the fit of each strategy and to compare them to each other. Therein, the difference between m experimental (*y*_*i*_) and simulated data ($\bar{y_{i}}$) is calculated on a logarithmic scale.

## Supporting information

S1 Text Supporting figures and tables. (PDF)
