# Supplementary material for: Model-based approach for predicting the impact of genetic modifications on product yield in biopharmaceutical manufacturing—Application to influenza vaccine production
Source: PLoS Comput Biol. 2020 Jun 29;16(6):e1007810. doi: 10.1371/journal.pcbi.1007810 (PMC7323952; doi:10.1371/journal.pcbi.1007810)
Supplement: S1 Text — (PDF) [file pcbi.1007810.s001.pdf]

S1 Text. Supporting figures and tables

Model-based approach for predicting the impact of genetic modifications on product yield in biopharmaceutical manufacturing - Application to influenza vaccine production

Stefanie Duvigneau[1,†], Robert Dürr[2,†,*], Tanja Laske[3,†], Mandy Bachmann[3], Melanie Dostert[3], Achim Kienle[1,2]

1 Institute for Automation Engineering, Otto von Guericke University, Magdeburg, Saxony-Anhalt, Germany
2 Process Synthesis and Process Dynamics, Max Planck Institute for Dynamics of Complex Technical Systems, Magdeburg, Saxony-Anhalt, Germany
3 Bioprocess Engineering, Max Planck Institute for Dynamics of Complex Technical Systems, Magdeburg, Saxony-Anhalt, Germany

* Corresponding author: duerr@mpi-magdeburg.mpg.de
† These authors contributed equally to this work

Table A. Fold overexpression level (FOE) of single gene overepressions in A549 cells after lentiviral transducion.

	CEACAM6	FANCG	NXF1	PLD2	XAB2
FOE	18.63	6.86	1.50	14.00	3.30

Table B. RMS values for the simulation with the SGO parameter distributions

	A549	EMCV	CEACAM6	FANCG	NXF1	PLD2	XAB2
RMS	0.58	0.54	0.52	0.36	0.32	0.49	0.50

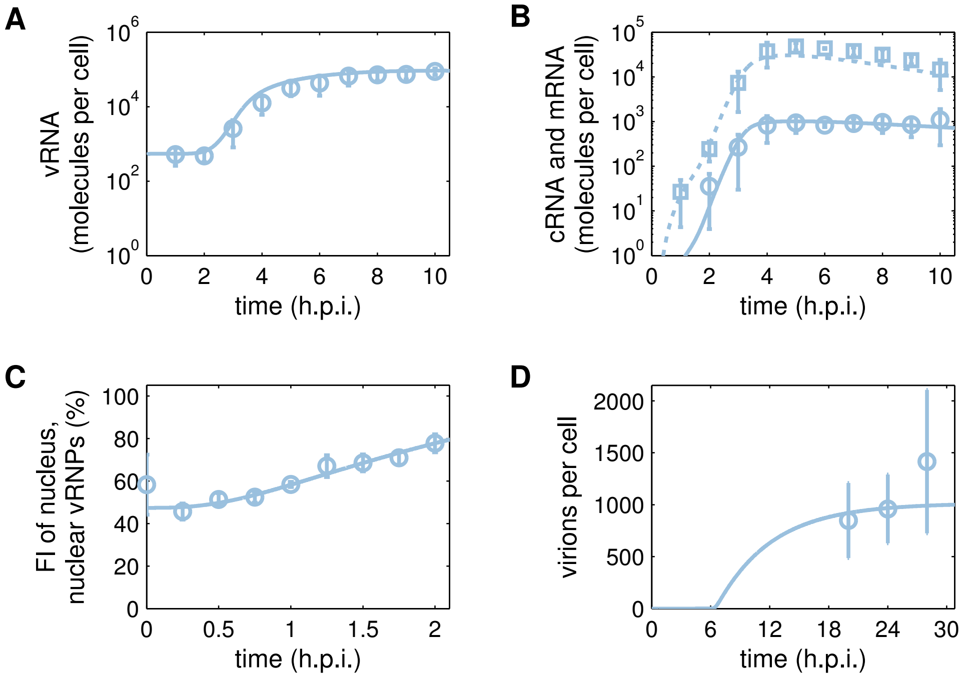

Fig A. Intracellular IAV replication and virus release of infected parental A549 cells. Fit of the intracellular model published in [1](blue lines) to experimental data (blue symbols) are shown for intracellular dynamics of (A) viral RNA as well as for (B) viral mRNA (squares, dashed line) and cRNA (circles, solid line) as measured by RT-qPCR for an infection at MOI 50. (C) The nuclear import of vRNPs in CHX-treated cells was assessed by imaging flow cytometry and the simulated fraction of nuclear RNPs was fitted to the relative fluorescence intensity (FI) of the nucleus upon infection at MOI 50. (D) The simulated release of viral progeny per cell was fitted to the cell-specific virus yield calculated based on the HA assay and the maximum viable cell count during an infection at MOI 1. For model simulation, kinetic parameters were set to the median of the bootstrap-parameter distributions acquired in the present study.

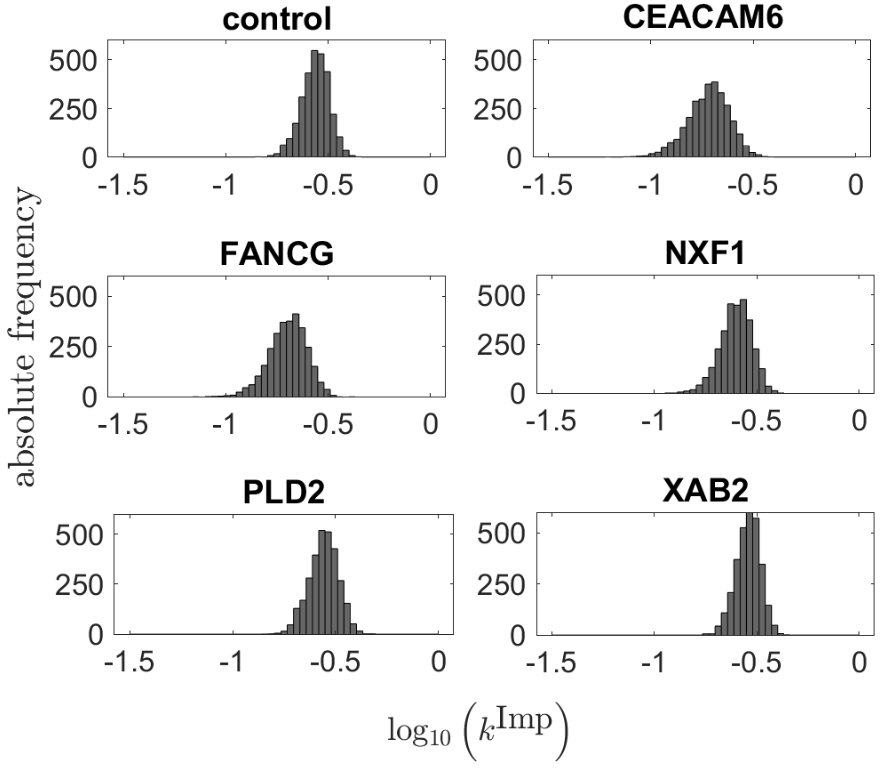

Fig B. Parameter distributions for the kinetic parameter $\log_{10}\left(\mathbf{k}^{\mathbf{Imp}}\right)$. Parameter distributions were estimated by parametric bootstrapping [2]. Multiple optimization runs were performed to fit 1000 randomly resampled measurement points.

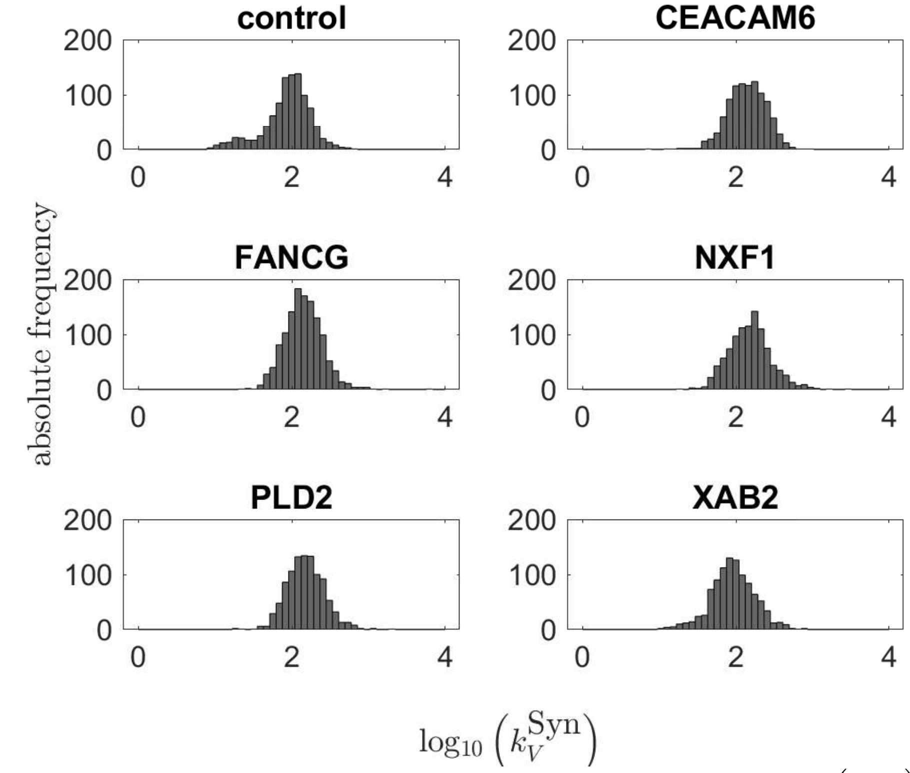

Fig C. Parameter distributions for the kinetic parameter $\log_{10}\left(\mathbf{k_V^{Syn}}\right)$.
Details for the determination of the distributions can be found in the caption of Fig B

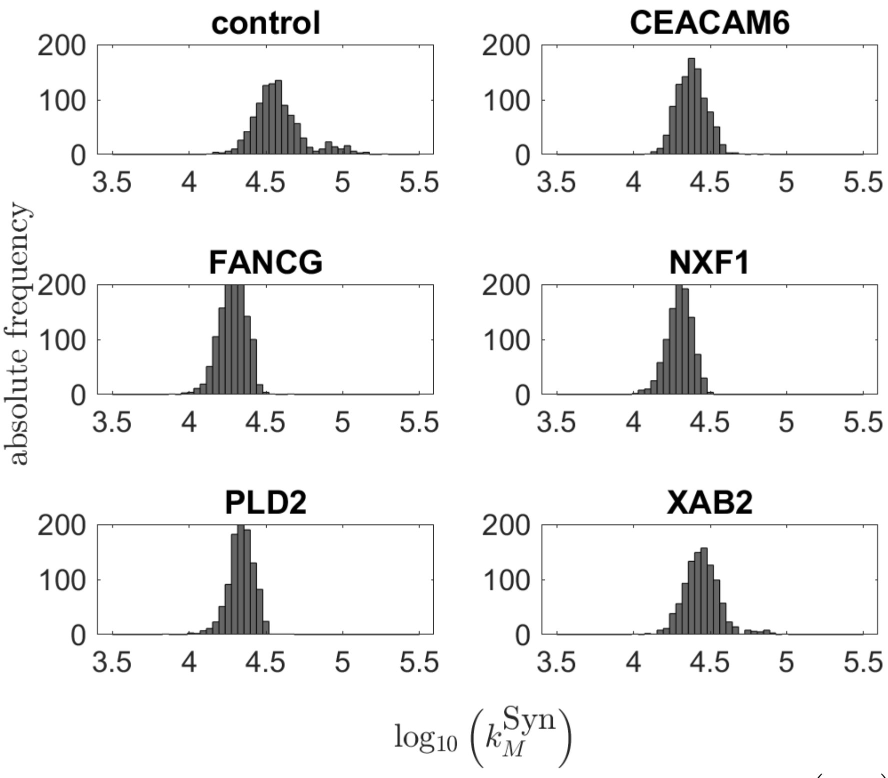

Fig D. Parameter distributions for the kinetic parameter $\log_{10}\left(\mathbf{k}_{\mathbf{M}}^{\mathbf{Syn}}\right)$. Details for the determination of the distributions can be found in the caption of Fig B

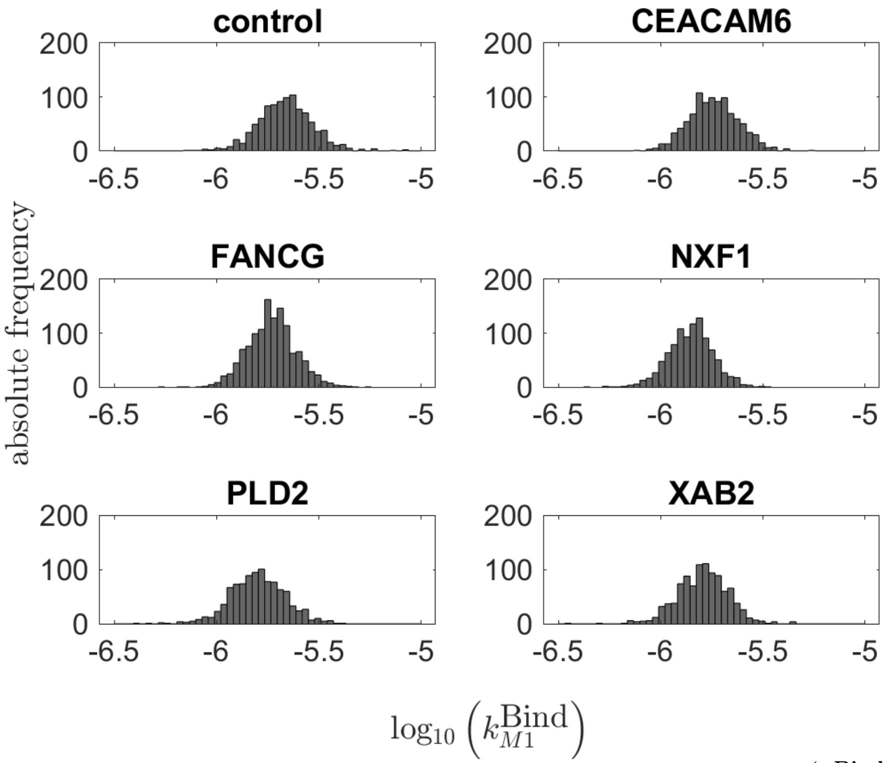

Fig E. Parameter distributions for the kinetic parameter $\log_{10}\left(\mathbf{k}_{\mathbf{M1}}^{\mathbf{Bind}}\right)$. Details for the determination of the distributions can be found in the caption of Fig B

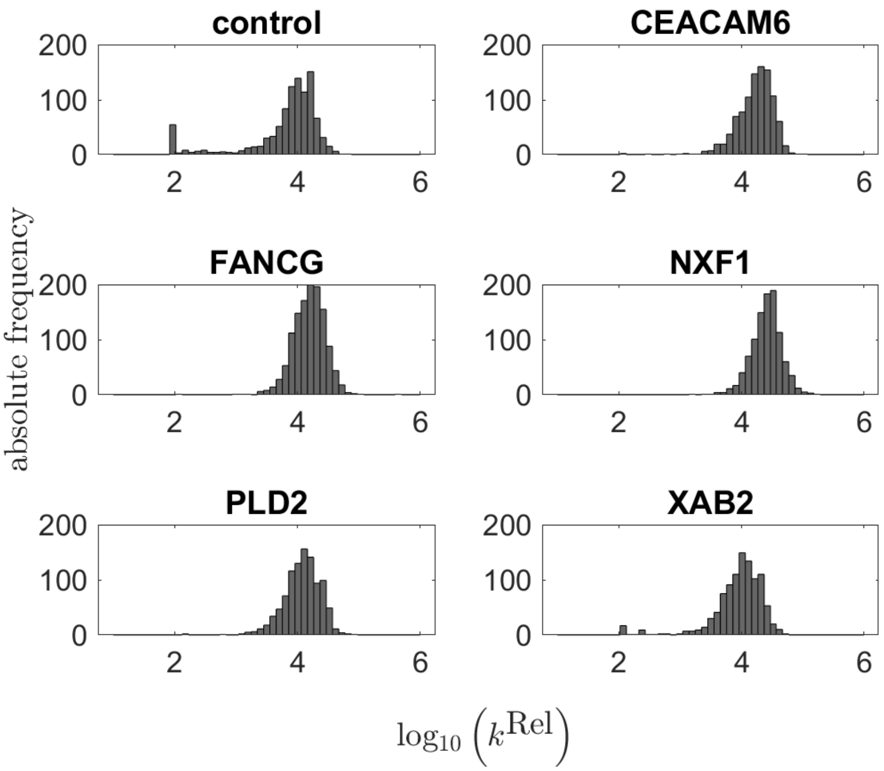

Fig F. Parameter distributions for the kinetic parameter $\log_{10}\left(\mathbf{k}^{\mathbf{Rel}}\right)$.
Details for the determination of the distributions can be found in the caption of Fig B

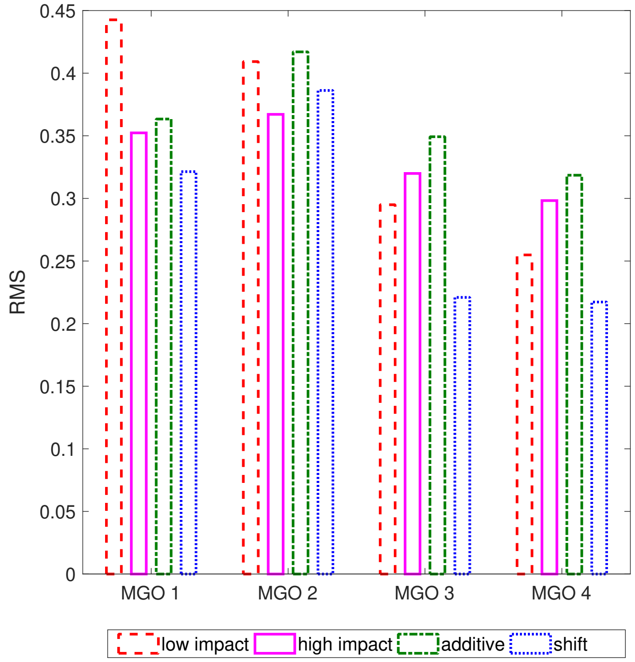

Fig G. RMS values for each MGO and strategy. The values for each strategy were calculated as described in the paragraph Strategy selection. The modified genes of each MGO are shown in Table 1.

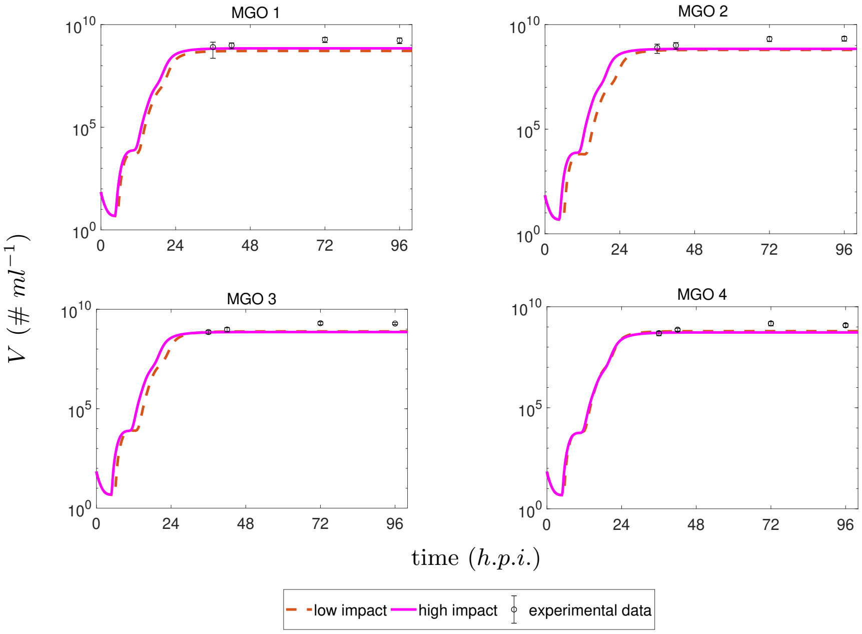

Fig H. Experimental data and model simulations for the low and high impact strategy. All validated MGOs were infected with an MOI of 10^{-4}. The modified genes of each MGO are shown in Table 1.

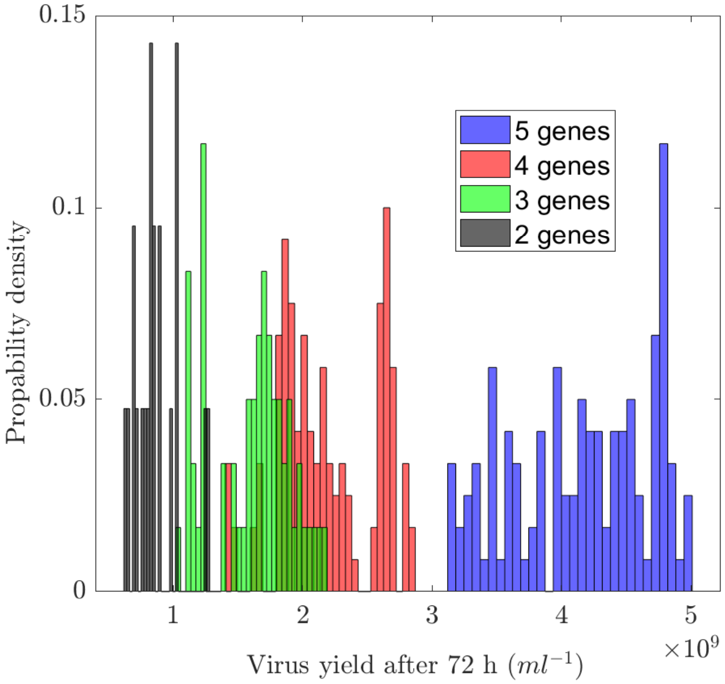

Fig I. Histogram of all MGO combinations sorted by the number of modified genes. The considered genes for modification are: CEACAM6, FANCG, NXF1, PLD2 and XAB2.

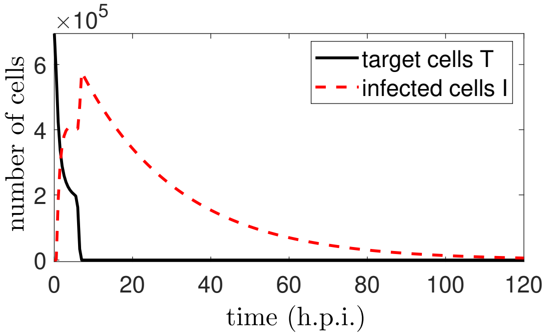

Fig J. Dynamics of targets and infected cells after infection at MOI 1. The distributions of the parental cell line (A549, control) were used for illustration.

Table C. Nomenclature

i_c	Number density distribution of infected cells
$\mathcal{I}(\mathbf{x})$	Distribution of newly infected cells
k_T^{Apo}	Natural apoptosis rate of uninfected cells
k_I^{Apo}	Apoptosis rate due to infection
h^*	Single cell dynamics of the extended state vector
$\mathbf{x}^*$	Extended state vector
r^{Inf}	Infection rate
T	Uninfected target cells
k^{Imp}	Import rate of virus particles
k_M^{Syn}	Synthesis rate of viral mRNA
k_C^{Syn}	Synthesis rate of viral cRNA
k_V^{Syn}	Synthesis rate of viral vRNA
k_{M1}^{Bind}	Binding rate of M1 to nuclear VRNPa
k^{Rel}	Release rate constant of virus particles
I_a	Apoptotic infected cells
k^{Lys}	Lysis rate constant of apoptotic cells
g	Growth rate
T_a	Apoptotic uninfected target cells
$g_{\max}$	Maximum growth rate
$T_{\max}$	Maximum uninfected target cells
V	Free virus particles
V_n^{Att}	Attached virus particles with low or high affinity
V^{En}	Virus particles in the endosomes of infected cells
r^{Rel}	Release rate of virus particles
k_V^{Deg}	Clearance rate of virus particles
k_n^{Dis}	Dissociation rates of virus particles with low or high affinity
$k_{c,n}^{\mathrm{Att}}$	Attachment rates of virus particles with low or high affinity
B_n	Number of high and low affinity binding sites
k^{En}	Endocytosis rate
r^{Lys}	Lysis rate
k^{Fus}	Fusion rate with endosomes
B_n^{tot}	Total number of high and low affinity binding sites
$k_{c,n}^{\mathrm{Equ}}$	Equilibrium constant of high and low affinity sites
n	Affinity of viral attachment, low or high
F_{inf}	Ratio of infected cells to fused virus particles
Vp_{M1}^{cyt}	vRNP-M1 complex in the cytoplasm
K_{Vrel}	Impact of viral components on release
$RdRp$	RdRp complexes in infected cells
P_j	Concentration of viral protein j
N_j	Amount of viral component j
j	Viral components
Rnp^{cyt}	Total vRNP in the cytoplasm
Rnp^{nuc}	Total vRNP in the nucleus
Vp^{cyt}	vRNP in the cytoplasm
Vp^{nuc}	vRNP in the nucleus
Vp_{M1}^{nuc}	vRNP-M1 complex in the nucleus
$frac_{\mathrm{Rnp}}^{\mathrm{nuc}}$	Simulated fraction of nuclear vRNPs
$frac_{\mathrm{Int}}^{\mathrm{nuc}}$	Relative fluorescence intensity of the nucleus
J	Objective function for the identification of k^{Imp}

$J^{\log}$	Objective function for the identification of $\left[k_M^{\text{Syn}} k_V^{\text{Syn}} k_C^{\text{Syn}} k_{M1}^{\text{Bind}} k^{\text{Rel}}\right]$
$k_{\{1,2,3,4,5\}}$	Gaussian distributions for the low and high impact strategy
$a_{\{1,2,3,4,5\}}$	Scaling factors for the gaussian distributions
$\mu_{\{1,2,3,4,5\}}$	Mean values for the gaussian distributions
$EL_{i,\text{Rel}}$	Relative frequency for SGO i
$\Delta_{i,\text{Rel}}$	Relative distance to the median value of the base and second SGO
FOE_i	Fold overexpression of SGO i
Δ_i	Distance to the median value of the base and second SGO
RMS	Root mean square
y_i	Experimental data point i
$\bar{y}_i$	Simulated data point i
m	Number of data points

References

1. Heldt FS, Frensing T, Pflugmacher A, Gröpler R, Peschel B, Reichl U. Multiscale Modeling of Influenza A Virus Infection Supports the Development of Direct-Acting Antivirals. PLoS Computational Biology. 2013;9(11).

2. Egea JA, Rodríguez-Fernández M, Banga JR, Martí R. Scatter search for chemical and bio-process optimization. Journal of Global Optimization. 2007;37(3):481–503.
